# Comparison of the Mapleson C circuit, 500 ml and 1.6 l self-inflating bags for delivering guideline-compliant ventilation during simulated adult cardiopulmonary resuscitation

**DOI:** 10.1186/cc9710

**Published:** 2011-03-11

**Authors:** P Sherren, A Lewinsohn, T Jovaisa, S Wijayatilake

**Affiliations:** 1 Queens Hospital, Romford, UK

## Introduction

Despite all the research and education that has gone into the field of cardiopulmonary resuscitation (CPR), survival rates remain bleak. A significant problem has been the discrepancy between teachings and witnessed clinical practice. As a result of this, and the deleterious outcomes associated with hyperventilation, we conducted a manikin-based study to evaluate three different ventilating devices and their ability to provide guideline-compliant ventilation during simulated adult CPR.

## Methods

A simulated cardiac arrest scenario was undertaken by 33 healthcare professionals (α = 0.05, power = 80%). Participants were asked to ventilate a simulated cardiac arrest patient for a period of 1 minute with all three devices, during which time various ventilatory parameters were recorded using a spirometer. The devices investigated were the Mapleson C circuit, adult (1.6 l) and paediatric (500 ml) self-inflating bags. *P *< 0.01 was deemed statistical significant, due to multiple comparisons.

## Results

The paediatric self-inflating bag performed best, with significant improvement in the mean minute ventilation (*P = *0.003), tidal volume (*P *< 0.001) and peak airway pressure (*P *< 0.001). Despite the significant differences, the paediatric self-inflating bag still delivered a mean minute ventilation of 7.01 l/minute, which still exceeds the Resuscitation Council's suggested 5 l/minute. See Table 1.

**Table 1:** Comparison of data on ventilation parameters

	500 ml self-inflating bag	1.6 l self-inflating bag	Mapleson C circuit	*P *value
Minute ventilation (l/minute)	7.01 (3.22)	9.68 (4.22)	9.77 (3.45)	0.003*
Tidal volume (nl)	391 (51.5)	582 (86.7)	625 (103)	< 0.001*
Respiratory rate (/minute)	16.7 (6.9)	18 (6.45)	17.3 (5.46)	0.704^†^
Peak airway pressure (cmH_2_O)	14.5 (5.18)	20.7 (9.03)	30.3 (11.4)	< 0.001*

Data presented as mean (SD). *Statistically significant result. ^†^Nonstatistically significant result.

## Conclusions

Participants were found to be hyperventilating simulated cardiac arrest patients with all devices. The paediatric self-inflating bags delivered the most guideline-compliant ventilation and its use in adult CPR may be a simple measure to ensure delivery of more guideline-consistent ventilation.

